# Session Coalgebras: A Coalgebraic View on Session Types and Communication Protocols

**DOI:** 10.1007/978-3-030-72019-3_14

**Published:** 2021-03-23

**Authors:** Alex C. Keizer, Henning Basold, Jorge A. Pérez

**Affiliations:** Imperial College, London, UK; 9 Master of Logic, ILLC, University of Amsterdam, Amsterdam, The Netherlands; 10 LIACS – Leiden University, Leiden, The Netherlands; 11 University of Groningen, Groningen, The Netherlands; 12 CWI, Amsterdam, The Netherlands

**Keywords:** Session types, Coalgebra, Process calculi, Coinduction.

## Abstract

Compositional methods are central to the development and verification of software systems. They allow breaking down large systems into smaller components, while enabling reasoning about the behaviour of the composed system. For concurrent and communicating systems, compositional techniques based on *behavioural type systems* have received much attention. By abstracting communication protocols as types, these type systems can statically check that programs interact with channels according to a certain protocol, whether the intended messages are exchanged in a certain order. In this paper, we put on our coalgebraic spectacles to investigate *session types*, a widely studied class of behavioural type systems. We provide a syntax-free description of session-based concurrency as states of coalgebras. As a result, we rediscover type equivalence, duality, and subtyping relations in terms of canonical coinductive presentations. In turn, this coinductive presentation makes it possible to elegantly derive a decidable type system with subtyping for $\pi$-calculus processes, in which the states of a coalgebra will serve as channel protocols. Going full circle, we exhibit a coalgebra structure on an existing session type system, and show that the relations and type system resulting from our coalgebraic perspective agree with the existing ones.

## 1 Introduction

Communication protocols enable interactions between humans and computers alike, yet different scientific communities rely on different descriptions of protocols: one community may use textual descriptions, another uses diagrams, and yet another may use types. There is then a mismatch, which is fruitful and hindering at the same time. Fruitful, because different views on protocols lead to different insights and technologies. But hindering, because exactly those insights and technologies cannot be easily exchanged. With this paper, we wish to provide a view of protocols that opens up new links between communities and that, at the same time, contributes new insights into the nature of communication protocols.

What would such a view of communication protocols be? Software systems typically consist of concurrent, interacting processes that pass messages over channels. Protocols are then a description of the possible exchanges on channels, without ever referring to the exact structure of the processes that use the channels. Since we may, for example, expect to get an answer only after sending a question, it is clear that such exchanges have to happen in an appropriate order. Therefore, protocols have to be a *state-based abstraction of communication behaviour* on channels. Because *coalgebras* provide an abstraction of general state-based behaviour, our proposed view of communication protocols becomes: model the states of a protocol as states of a coalgebra and let the coalgebra govern the exchanges that may happen at each state of the protocol.

The above view of protocols allows us to model protocols as coalgebras. However, protocols are usually not studied for the sake of their description but to achieve certain goals: ensuring correct composition of processes, comparing communication behaviour, or refining and abstracting protocols. *Session types* [19,20] are an approach to communication correctness for processes that pass messages along channels. The idea is simple: describe a protocol as a syntactic object (a type), and use a type system to statically verify that processes adhere to the protocol. This syntactic approach allows the automatic and efficient verification of many correctness properties. However, the syntactic approach depends on choosing *one particular representation of protocols* and *one particular representation of processes*. We show in this paper that our coalgebraic view of protocols can guarantee correct process composition, and allows us to reason about key notions in the world of session types, *type equivalence*, *duality* and *subtyping*, while being completely independent of protocol and process representations.

Our coalgebraic view is best understood by following the distillation process of ideas on a concrete session type system by Vasconcelos [37]. Consider the session type $S = ?\texttt{int}.\,!\texttt{bool}.\,\text{end}$, which specifies the protocol on one endpoint of a channel that receives an integer, then outputs a Boolean, and finally terminates the interaction. Note that the protocol $S$ specifies three different states: an input state, an output state, and a final state. Moreover, we note that $S$ specifies only how the channel is seen from one endpoint; the other endpoint needs to use the channel with the *dual* protocol $!\texttt{int}.\,?\texttt{bool}.\,\text{end}$. Thus, session type systems ensure that the states of $S$ are enabled only in the specified order and that the two channel endpoints implement dual protocols.

A state-based reading of session types is intuitive and is already present in programming concepts such as typestates [15,32,33], theories of behavioural contracts [4,6,7,13], and connections between session types and communicating automata [10,25]. The novelty and insight of the coalgebraic view is that 1. it describes the state-based behaviour of protocols underlying session types, supporting unrestricted types and delegation, without adhering to any specific syntax or target programming model; 2. it offers a general framework in which key notions such as type equivalence, duality, and subtyping arise as instances of well-known coinductive constructions; and 3. it allows us to derive type systems for specific process languages, like the $\pi$-calculus.

*Session Coalgebras at Work* How does this coalgebraic view of protocols work for general session types? Consider a "mathematical server" that offers three operations to clients: integer multiplication, Boolean negation and quitting. The following session type $T$ specifies a protocol to communicate with this server.

$$T = \mu X. \; \& \begin{cases} \mathit{mul}: & ?\texttt{int}.\ ?\texttt{int}.\ !\texttt{int}.\ X \\ \mathit{neg}: & ?\texttt{bool}.\ !\texttt{bool}.\ X \\ \mathit{quit}: & \mathrm{end} \end{cases}$$

$T$ is a recursive protocol, as indicated by "$\mu X.$", which can be repeated. A client can choose, as indicated by &, between the three operations (*mul*, *neg*, and *quit*) and the protocol then continues with the corresponding actions. For instance, after choosing *mul*, the server requests two integers and, once received, promises to send an integer over the channel. We can see states of the protocol $T$ emerging, and it remains to provide a coalgebraic view on the actions of the protocol to obtain what we will call *session coalgebras*.

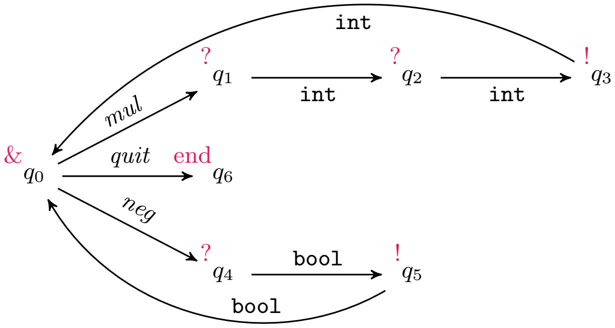

**Figure 1.** Protocol of the mathematical server as a session coalgebra

Fig. 1 depicts a session coalgebra that describes protocol $T$. It consists of states $q_0, \ldots, q_6$, each representing a different state of $T$, and transitions between these states to model the evolution of $T$. Meaning is given to the different states and transitions through the labels on the states and transitions. The state *labels*, written in purple at top-left of the state name, indicate the branching type of that state. Depending on the branching type, the labels of the transitions bear different meanings. For instance, $q_0$ is labelled with "&", which indicates that this state initiates an external choice. The labels on the three outgoing transitions for $q_0$ (*mul*, *neg*, *quit*) correspond then to the possible kinds of message for selecting one of the branches. Continuing, states $q_1, \ldots, q_5$ are labelled with a request for data (label ?) or the sending of data (label !), and the outgoing transition labels indicate the type of the exchanged values (e.g., `bool`). Finally, state $q_6$ decrees the end of the protocol. Note that the cyclic character of $T$ occurs as transitions back to $q_0$; there is no need for an explicit operator to capture recursion.

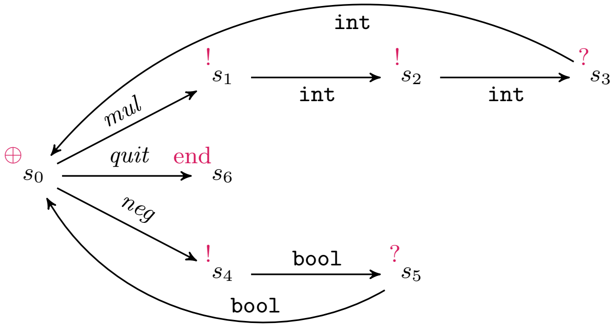

**Figure 2.** Session coalgebra for the client view protocol the of mathematical server

A session coalgebra models the view on one channel endpoint, but to correctly execute a protocol we also need to consider the *dual* session coalgebra that models the other endpoint's view. In our example, the dual of Fig. 1 is given by the diagram in Fig. 2, which concerns states $s_0, \ldots, s_6$. More precisely, the *states* $q_i$ and $s_i$ are pairwise dual in the following sense. The external choice of $q_0$ becomes an *internal choice* for $s_0$, expressed through the label $\oplus$, with exactly the same labels on the transitions leaving $s_0$. This means that whenever the server's protocol is in state $q_0$ and the client's protocol in state $s_0$, then the client can choose to send one of the three signals to the server, thereby forcing the server protocol to advance to the corresponding state. All other states turn from sending states into receiving states and vice versa. We will see that this *duality relation* between states of session coalgebras has a natural coinductive description that can be obtained with the same techniques as bisimilarity. The duality relation for $T$ will give us then the full picture of the intended protocol.

Suppose a client who would only want to use multiplication once but could also handle real numbers as inputs. Such a client had to follow the protocol given by the session coalgebra in Fig. 3, with states $r_0, \ldots, r_5$.

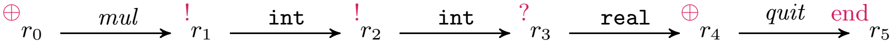

**Figure 3.** Session coalgebra that uses only part of a mathematical server

In theories of session types, the protocol of Fig. 2 would be a *subtype* of this one (cf. [17,16]). Concretely, this new client can also follow the subtype protocol, and can thus communicate with a server following the protocol of Fig. 1. For session coalgebras, we recover the same notion of subtyping by using specific *simulation* relations that allow us to prove that the behaviour of $r_0$ can be simulated by $s_0$. Together, simulations and duality provide the foundation of typical session type systems.

We have used thus far session types and coalgebras for protocols with simple control and with exchanges of simple data values. In contrast, rich session type systems can regulate *session delegation*, the dynamic allocation and exchange of channels by processes. Imagine a process that creates a channel, which should adhere to some protocol $T$. From an abstract perspective, the process holds both endpoints of the new channel, and has to send one of them to the process it wishes to communicate with. To ensure statically that the receiving process respects the protocol of this new channel, we need to announce this communication as a transmission of the session type $T$ (via an existing channel) and use $T$ to verify the receiving process. Session delegation adds expressiveness and flexibility, but may cause problems in the characterisation of a correct notion of duality [18]. Remarkably, our coalgebraic view of session types makes this characterisation completely natural.

As an example, consider the type $T = \mu X.\ ?X.\ X$, which models a channel endpoint that infinitely often receives channel ends of its own type $T$. To obtain the dual of $T$, we may naïvely try to replace the receive with a send, which results in the type $\mu X.\ !X.\ X$. The problem is that the two channel endpoints would not agree on the type they are sending or receiving, as any dual type of $T$ needs to send messages of type $T$. Thus, the correct dual of $T$ would be the type $U = \mu X.\ !T.\ X$. Both $T$ and $U$ specify the transmission of non-basic types, either the recursion variable $X$ or $T$, in contrast to the mathematical server that merely stipulated the transmission of basic data values (integers or Booleans).

In our session coalgebras for the mathematical server it sufficed to have simple data types and branching labels on transitions. However, to represent $T$ and $U$ we will need another mechanism to express session delegation. We observe that a transmission in session types consists of the transmitted data and the session type that the protocol must continue with afterwards. Thus, a transition out of a transmitting state in a session coalgebra encompasses both a *data transition* and a *continuation transition*. In diagrams of session coalgebras, we indicate the data transition by a coloured arrow ⟶ and an arrow ⇢ connecting the data to the continuation transition. Using the combined transitions, Fig. 4 redraws the multiplication part of the mathematical server in Fig. 1.

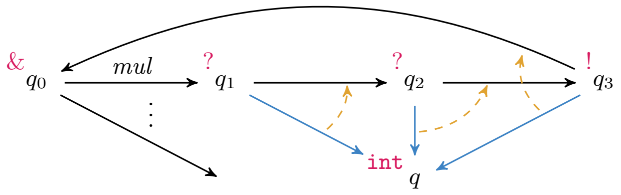

**Figure 4.** Protocol of mathematical server as session coalgebra

This way, the transition $q_1 \xrightarrow{\texttt{int}} q_2$ has been replaced by *both* a data transition into a new state $q$ *and* a continuation transition into $q_2$. Moreover, $q$ has been declared as a *data state* that expects an integer to be exchanged (label `int`).

Having added these transitions to our toolbox, we can present the two types $T$ and $U$ as session coalgebras. The diagram in Fig. 5 shows such a session coalgebra, in which we name the states suggestively $T$ and $U$.

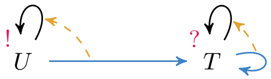

**Figure 5.** Session coalgebra for a recursive type $T$ and its dual $U$

Using this presentation as session coalgebras, it is now straightforward to *coinductively* prove that the states $T$ and $U$ are dual: 1. the states have opposite actions; 2. their data transitions point to equal types; and 3. their continuations are dual by coinduction. Clearly, the last step needs some justification but it will turn out that we can appeal to a standard definition of coinduction in terms of greatest fixed points. This demonstrates that our coalgebraic view on session types makes the definition of duality truly natural and straightforward.

Up to here, we have discussed session types and coalgebras that are *linear*, i.e., they enforce that protocols complete exactly once. In many situations, one also needs *unrestricted* types, which enable sharing of channels between processes that access these channels concurrently. This is the case of a process that offers a service for other processes, for instance a web server. Session delegation allows us to create dynamically channels and check their protocols, but the shared channel for *initiating a session* [17] has to offer its protocol to an arbitrary number of clients. Unrestricted types enable us to specify these kind of service offers.

As an example, consider a process that provides a channel for communicating integers to anyone asking, like a town hall official handing out citizen numbers. The type $U' = \mu X.\, \texttt{un}\,!\texttt{int}.\, X$ represents the corresponding protocol, where "un" qualifies the type $!\texttt{int}.\, X$ as unrestricted. This allows the process holding the end of a channel with type $U'$ to transmit an integer to any process that is connected to the shared channel, without any restriction on their number. It is now surprisingly simple to express $U'$ in our coalgebraic view: we introduce a new state label "un" (unrestricted), which expresses that states reachable from this state can be used arbitrarily as protocols across different processes connecting to a channel that follow the protocol given by those states. The following diagram shows a session coalgebra with a state that corresponds to the type $U'$.

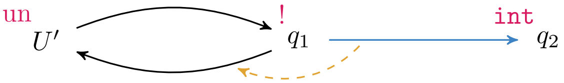

*Contributions and Related Work.* In this paper, we introduce the notion of *session coalgebra*, which justifies the state-based behaviour of session types from a coalgebraic perspective. This perspective is novel, although specific state-based description of protocols have been considered before [4,6,7,9,10,13,15,25,32,33]. Using coalgebra as a unifying framework for session types has two advantages: 1. session coalgebras can be defined and studied independently from specific syntactic formulations, while keeping the operational behaviour of session types; and 2. we can uncover the innate *coinductive* nature of key notions in session types, such as duality, subtyping, and type equivalence through standard coalgebraic techniques. In particular, although communicating automata can also provide syntax-independent characterisations of session types [10,11], such characterisations do not support delegation, an expressive feature which is cleanly justified in our coalgebraic approach. Coinduction already has been exploited in the definition of type equivalence [35], subtyping [17,16] and, especially, duality for systems with recursive types [3,18,24]. Unlike ours, these previous definitions are language-dependent, as they are tailored to specific process languages and/or syntactic variants of the type discipline. Session coalgebras enable thus the generalisation of insights and technologies from specific languages to any protocol specification that fits under the umbrella of state-based sessions.

To enable the verification of processes against protocols described by session coalgebras, we also contribute a *type system* for $\pi$-calculus processes, in which channel types are given by states of an *arbitrary session coalgebra*. Our type system revisits the one by Vasconcelos [37] from our coalgebraic perspective, while extending it with subtyping. Moreover, we provide a *type checking algorithm* for that system, provided that the underlying session coalgebra fulfils two intuitive conditions. In doing so, we show how a specific type syntax can be equipped with a session coalgebra structure and how the two decidability conditions are reflected in the type system. This is in contrast to starting with a specific type syntax and then employing category theoretical ideas [36], where coinductive session types are encoded in a session type system with parametric polymorphism [5]. Instead, we show how a session type system can be derived in general from coalgebras.

*Organisation* Throughout the remaining paper we will turn the sketched ideas into a coalgebraic framework. We introduce in Sec. 2 a concrete session type syntax that we will use as illustration of our framework. In Sec. 3, we will define session coalgebras as coalgebras for an appropriate functor and show that the type system from Sec. 2 can be equipped with a coalgebraic structure. The promised coinductive view on type equivalence, duality, subtyping, etc. will be provided in Sec. 4. Moreover, we will show that these notions are decidable under certain conditions that hold for any reasonable session type syntax, including the one from Sec. 2. Up to that point, the session coalgebras only had intrinsic meaning and were not associated to any process representation. Section 5 sets forth a type system for $\pi$-calculus, in which channels are assigned states of a session coalgebra as types. The resulting type system features subtyping and algorithmic type checking, presented in Sec. 6. Some final thoughts are gathered in Sec. 7. An extended version, available online, collects additional material [22].

$$\begin{array}{llll} p ::= ?T.\, T & & T ::= d \in D \\ \quad \mid\ !T.\, T & & \quad \mid\ \mathsf{end} \\ \quad \mid\ \&\{l_i : T_i\}_{i \in I} & & \quad \mid\ q\, p \\ \quad \mid\ \oplus\{l_i : T_i\}_{i \in I} & & \quad \mid\ X \in \mathsf{Var} \\ & & \quad \mid\ \mu X.T \\ q ::= \mathsf{lin} \mid \mathsf{un} & & \end{array}$$

**Figure 6.** Session types over sets of basic data types $D$ and of variables $\mathsf{Var}$

## 2 Session Types

To motivate the development of session coalgebras, we recall in this section the concrete syntax of an existing session type system by Vasconcelos [37]. After building up our intuition, we introduce session coalgebras in Sec. 3 to show they can represent this concrete type system.

The types of the system that we will be using are generated by the grammar in Fig. 6, relative to a set of basic data types $D$ and a countable set of type variables $\mathsf{Var}$. This grammar has three syntactic categories: pretypes $p$, qualifiers $q$, and session types $T$. A pretype $p$ is simply a communication action: send (!), receive (?), external choice (&), and internal choice ($\oplus$) indexed by a finite sets $I$ of labels, followed by one or multiple session types. The simplest session types are basic data types in $D$ and the completed, or terminated, protocol represented by end. A pretype and qualifier also form a session type, written as $q\ p$. The "$\mathsf{lin}$" qualifier enforces that the communication action $p$ has to be carried out exactly once, while the "$\mathsf{un}$" qualifier allows arbitrary use of $p$. Finally, we can form recursive session types with the the fixed point operator $\mu$ and the use of type variables. We use the usual notion of $\alpha$-equivalence, (capture-avoiding) substitution, and free and bound types variables for session types.

The grammar allows arbitrary recursive types. We let $\mathsf{Type}$ be the set of all $T$ in which recursive types are *contractive* and *closed*, which means that they contain no substrings of the form $\mu X_1.\mu X_2 \ldots \mu X_n.X_i$ and no free type variables.

To lighten up notation, we will usually omit the qualifier $\mathsf{lin}$ and assume every type to finalise with end. With these conventions, we write, e.g., ?`int`. instead of $\mathsf{lin}$ ?`int`. end and $\mathsf{un}$ ?`int`. for a single unrestricted read.

We assume there is some decidable subtyping preorder $\leq_D$ over the basic types. A type is a subtype of another if the subtype can be used anywhere where the supertype was accepted. In examples, we use the basic types `int`, `real` and `bool`, and we assume that `int` is a subtype of `real`, as usual.

An important notion is the *unfolding* of a session type, which we define next:

**Definition 1 (Unfolding).** *The unfolding of a recursive type $\mu X.T$ is defined recursively*

$$\mathit{unfold}(\mu X.T) = \mathit{unfold}(T[\mu X.T/X])$$

*For all other $T$ in* $\mathsf{Type}$*, unfold is the identity: $\mathit{unfold}(T) = T$.*

Because we assume that types are contractive, $\mathit{unfold}(T)$ terminates for all $T$. Also, because all types are required to be closed, $\mathit{unfold}(T)$ can never be a variable $X$. Any such variable would have to be bound somewhere before use, meaning it would have been substituted. Furthermore, unfolding a closed type always yields another closed type, as each removed binder always causes a substitution of the bound variable.

## 3 Session Coalgebra

Here we will discuss *session coalgebras*, the main contribution of this paper. The idea is that session coalgebras will be coalgebras for a specific functor $F$, which will capture the state labels and the various kinds of transitions that we discussed in Sec. 1. An important feature of coalgebras in general, and session coalgebras in particular, is that the states can be given by an arbitrary set. We will leverage this to define a session coalgebra on the set of types $\mathsf{Type}$ introduced in Sec. 2.

Before coming to the definition, let us briefly recall some minimal notions of category theory. We will not require a lot of category theoretical terminology; in fact, we will only use the category **Set** of sets and functions. Moreover, we will be dealing with *functors* $F\colon \mathbf{Set} \to \mathbf{Set}$ on the category **Set**. Such a functor allows us to map a set $X$ to a set $F(X)$, and functions $f : X \to Y$ to functions $F(f) : F(X) \to F(Y)$. To be meaningful, a functor must preserve identity and compositions. That is, $F$ maps the identity function $\mathrm{id}_X\colon X \to X$ on $X$ to the identity on $F(X)$: $F(\mathrm{id}_X) = \mathrm{id}_{F(X)}$; and, given functions $f\colon X \to Y$ and $g\colon Y \to Z$, we must have $F(g \circ f) = F(g) \circ F(f)$.

A central notion is that of the *coalgebras* for a functor $F$. A coalgebra is given by a pair $(X, c)$ of a set $X$ and a function $c\colon X \to F(X)$. For simplicity, we often leave out $X$ and refer to $c$ as the coalgebra. The general idea is that the set $X$ is the set of *states* and that $c$ assigns to every state its one-step behaviour. In the case of session coalgebras this will be the state labels and outgoing transitions. Given two coalgebras $c\colon X \to F(X)$ and $d\colon Y \to F(Y)$, we say that $h\colon X \to Y$ is a *homomorphism*, if $d \circ h = F(h) \circ c$. Coalgebras and their homomorphisms form a category, with the same identity maps and composition as in **Set**.

We will have to analyse subsets of coalgebras that are closed under transitions. Given a coalgebra $c\colon X \to F(X)$, we say that $d\colon Y \to F(Y)$ with $Y \subseteq X$ is a *subcoalgebra* of $c$ if the inclusion map $Y \to X$ is a coalgebra homomorphism. Note that in this case $c(Y) \subseteq F(Y)$ and thus $d$ is the restriction of $c$ to $Y$. Hence, we also refer to $Y$ as subcoalgebra. The subcoalgebra *generated by* $x \in X$ in $c$, denoted by $\langle x \rangle_c$, is the least subset of $X$ that contains $x$ and is a subcoalgebra of $c$. Intuitively, it is the set of $x$ and all states that are reachable from $x$.

Coming to the concrete case of session coalgebras, we now construct a functor that allows us to capture the state labels and the different kinds of transitions. Keeping in mind that states of a session coalgebra correspond to states of a protocol, we need to be able to label the states with enabled operations.

**Definition 2 (Operations and Polarities).** *The* operation *of a state describes the action it represents:* com *marks the transmission (sending or receiving) of a*

*value;* branch *marks an (internal or external) choice;* end *marks the completed protocol;* bsc *marks a basic data type; and* un *marks an unrestricted type. States that transmit data (labelled with* com*) or allow for choice (labelled with* branch*) also have a* polarity*, which can be either* in *(a receiving action or external choice) or* out *(a sending action or internal choice). We let $O$ be the set of all operations $O = \{\mathrm{com}, \mathrm{branch}, \mathrm{end}, \mathrm{bsc}, \mathrm{un}\}$ and $P$ the set of polarities $P = \{\mathrm{in}, \mathrm{out}\}$.*

Note that pairs in $\{\mathrm{com}, \mathrm{branch}\} \times P$ directly correspond to the actions of a session type: $? = (\mathrm{com}, \mathrm{in})$, $! = (\mathrm{com}, \mathrm{out})$, $\& = (\mathrm{branch}, \mathrm{in})$ and $\oplus = (\mathrm{branch}, \mathrm{out})$. We will be using these markers to abbreviate the pairs.

Now that we have the possible operations of a protocol, we need to define the transitions that may follow each operation. Recall that the transition at a choice state has to be labelled with messages that resolve that choice. We therefore assume to be given a set $\mathbb{L}$ of possible choice labels. The variable $l$ will be used to refer to an element of $\mathbb{L}$. $\mathcal{P}^{+}_{<\aleph_0}(\mathbb{L})$ is the set of all finite, non-empty, subsets of $\mathbb{L}$. Variables $L, L_1, L_2, \ldots$ refer to these finite, non-empty subsets of $\mathbb{L}$.

Our goal is to define a *polynomial functor* [14] that captures the states labels and transitions. This requires some further formal language. First, we let $*$ and $\cancel{d}$ be some fixed, distinct, objects. Second, given sets $X$ and $Y$, we denote by $X^Y$ the set of all (total) functions from $Y$ to $X$. Finally, given a family of sets $\{X_i\}_{i \in I}$ indexed by some set $I$, their *coproduct* is the set $\coprod_{i \in I} X_i = \{(i, x) \mid i \in I, x \in X_i\}$.

We are now ready to define session coalgebras:

**Definition 3 (Session Coalgebras).** *Let $A$ and $B$ be sets defined as follows, where we recall that $D$ is the set of all basic data types.*

$$\begin{aligned} A = {} & \{\mathrm{com}\} \times P & B_{\mathrm{com},p} &= \{*, \cancel{d}\} \\ & \cup \{\mathrm{branch}\} \times P \times \mathcal{P}^{+}_{<\aleph_0}(\mathbb{L}) & B_{\mathrm{branch},p,L} &= L \\ & \cup \{\mathrm{end}\} & B_{\mathrm{end}} &= \emptyset \\ & \cup \{\mathrm{bsc}\} \times D & B_{\mathrm{bsc},d} &= \emptyset \\ & \cup \{\mathrm{un}\} & B_{\mathrm{un}} &= \{*\} \end{aligned}$$

*The polynomial functor $F : \mathbf{Set} \to \mathbf{Set}$ is defined by*

$$F(X) = \coprod_{a \in A} X^{B_a}$$

$$F(f)(a, g) = (a, f \circ g)$$

*A coalgebra $(X, c)$ for the functor $F$ is called a* session coalgebra*.*

Let us unfold this definition. Given a session coalgebra $c \colon X \to F(X)$ and a state $x \in X$, we find in $c(x) \in F(X)$ the information of $x$ encoded as a tuple $(a, f)$ with $a \in A$ and $f \colon B_a \to X$. From $a$, we get directly the operation, and the polarity for com states, the type of values communicated for bsc states or the message labels of branch states. The function $f$ encodes the transitions out of $x$. The domain of $f$ is exactly the set of labels that have a transition, and is dependent on the kind of state declared by $a$.

It is convenient to partition the domain of the transition map $f$ into data and continuations. Notice how only com states have data transitions, for other states, all transitions are continuations. As usual, we write $\mathrm{dom}(f)$ for the domain of $f$.

**Definition 4 (Domains).** *Suppose* $c(x) = (\text{com}, p, f)$*, then the* data domain *of* $f$ *is* $dom_D(f) = \{d\}$ *and the* continuation domain *is* $dom_C(f) = \{*\}$*. In all other cases,* $dom_D(f) = \emptyset$ *and* $dom_C(f) = dom(f)$*.*

### 3.1 Alternative Presentation of Session Coalgebras

Session coalgebras $(X, c)$ are rather complex. We show how to build up $c$ as the combination of two simpler functions, denoted $\sigma$ and $\delta$, so that $c(x) = (\sigma(x), \delta(x))$ with $\sigma\colon X \to A$ and $\delta(x)\colon B_{\sigma(x)} \to X$. Observe that every state gets an operation in $O$ assigned, thus we may assume that there is a map $\text{op}\colon X \to O$. Depending on the operation given by $\text{op}(x)$, the label on $x$ will then have different other ingredients that are captured in the following proposition.

To formulate the proposition, we need some notation. Suppose $f\colon X \to I$ is a map and $i \in I$. We define the *fibre* $X_i^f$ of $f$ over $i$ to be $X_i^f = \{x \in X \mid f(x) = i\}$. Moreover, we let the *pairing of functions* $f$ and $g$ be $\langle f, g\rangle(x) = (f(x), g(x))$.

**Proposition 1.** *A session coalgebra* $(X, c)$ *can equivalently be expressed by providing the following maps:*

$$\begin{aligned}
op &: X \to O && \textit{maps each state to an operation}\\
pol &: X^{op}_{\text{com}} + X^{op}_{\text{branch}} \to P && \textit{maps } \text{com} \textit{ and } \text{branch} \textit{ states to a polarity}\\
la &: X^{op}_{\text{branch}} \to \mathcal{P}^{+}_{<\aleph_0}(\mathbb{L}) && \textit{maps } \text{branch} \textit{ states to a set of labels}\\
da &: X^{op}_{\text{bsc}} \to D && \textit{maps } \text{bsc} \textit{ states to their basic type}\\
\delta_a &: X^{\sigma}_{a} \to X^{B_a} && \textit{maps each state to a transition function,}
\end{aligned}$$

*where*

$$\sigma(x) = \begin{cases} \langle op, pol\rangle(x) & \textit{if } op(x) = \text{com}\\ \langle op, pol, la\rangle(x) & \textit{if } op(x) = \text{branch}\\ \langle op, da\rangle(x) & \textit{if } op(x) = \text{bsc}\\ op(x) & \textit{if } op(x) = \text{end} \textit{ or } op(x) = \text{un} \end{cases}$$

We specified $\delta_a$ as a family of transition functions to preserve each specific signature. We can define a single global transition function as $\delta(x) = \delta_{\sigma(x)}(x)$. This is how the coalgebra finally becomes $c(x) = (\sigma(x), \delta(x))$. As long as the provided maps fit their signatures, this derived function will conform to $c : X \to F(X)$.

The procedure also works backwards: given any session coalgebra, we can derive functions $\text{op}(x)$, $\text{pol}(x)$, etc. from $c(x)$. We will often use $\text{op}(x)$, $\sigma(x)$, and $\delta(x)$ to refer to those specific parts of an arbitrary session coalgebra.

### 3.2 Coalgebra of Session Types

In Sec. 1, we informally explained how session types can be represented as states of a session coalgebra. We will now justify this claim by showing that session types are, in fact, states of a specific session coalgebra $(\mathsf{Type}, c_{\mathsf{Type}})$.

We define the functions op, pol, $\delta$, and la (see Prop. 1) on $\mathsf{Type}$. Using Prop. 1, we can then derive $c_{\mathsf{Type}} : \mathsf{Type} \to F(\mathsf{Type})$. Let us begin with the linear types.

| $T$ | $c_{\mathsf{Type}}(T)$ | | | |
|---|---|---|---|---|
| | op$(T)$ | pol$(T)$ | $\delta(T)$ | la$(T)$ |
| $\mathsf{lin}\,?T.\,T'$ | com | *in* | $\delta(T)(*) = T'$ | |
| $\mathsf{lin}\,!T.\,T'$ | | *out* | $\delta(T)(d) = T$ | |
| $\mathsf{lin}\&\{l_i : T_i\}_{i\in I}$ | branch | *in* | $\delta(T)(l_i) = T_i$ | $\{l_i \mid i \in I\}$ |
| $\mathsf{lin}\oplus\{l_i : T_i\}_{i\in I}$ | | *out* | | |

Under this definition, la$(T)$ is indeed finite, by virtue of an expression being a finite string. The completed protocol end and basic types $d$ are straightforward: $c(\text{end}) = (\text{end})$ and $c(d) = (\text{bsc}, d)$ for any $d \in D$. Recursive types are handled according to their unfolding, $c(\mu X.\,T) = c(\mathit{unfold}(\mu X.\,T))$. Recall that contractivity ensures that *unfold* always terminates. As our types are closed, all recursion variables are substituted during the unfolding of their binder. Consequently, we do not need to define $c$ on these variables. Also note that this definition results in an *equi-recursive* interpretation of recursive types.

Session types can also be unrestricted, and consist of a pretype $p$ with a qualifier $\mathsf{un}$. Session coalgebras have un states to mark unrestricted types; the continuation describes what the actual interaction is. Thus, we define op$(\mathsf{un}\ p) =$ un and $\delta(\mathsf{un}\ p)(*) = \mathsf{lin}\ p$.

*Remark 1 (Alternative Syntaxes and their Functors).* The unrestricted session types that we have adopted are fairly standard, but they are not the only ones in the literature. Most notably, Gay and Hole [17] defined a type $\frown[T_1, \ldots, T_n]$ that allows infinite reading *and* writing. To allow for such behaviour in session coalgebra, we can change $B_{\text{un}}$ to a set of two elements, such a $\{*_1, *_2\}$. Like internal choice, the two transitions describe an option of which behaviour to follow, but without sending synchronisation signals. One transition could go to a read, and the other to a write, both recursively continuing as the original type $\frown[T_1, \ldots, T_n]$.

It is possible, although not entirely trivial, to change the further definitions appropriately and get a decidable type checking algorithm encompassing both the syntax presented in this work, and Gay and Hole's syntax. We choose not to, so to keep the presentation simpler.

## 4 Type Equivalence, Duality and Subtyping

Up to here, we have represented session types as session coalgebras, but we have not yet given a precise semantics to them. As a first step, we will define three relations on states: *bisimulation*, *duality*, and *simulation*. Bisimulation is also called behavioural equivalence for types; we will show that bisimilar types are indeed equivalent. Duality specifies complementary types: it tells us which types can form a correct interaction. Simulation will provide a notion of subtyping: it tells us when a type can be used where another type was expected. Besides relations on session coalgebras, we also introduce the *parallelizability* of states that allows us to rule out certain troubling unrestricted types. Finally, we will obtain conditions on coalgebras to ensure the decidability of the three relations and therefore the type system that we derive in Sec. 5.

In the following, we will denote by $\mathrm{Rel}_X$ the poset $\mathcal{P}(X \times X)$ of all relations on $X$ ordered by inclusion. Recall that a post-fixpoint of a monotone map $g \colon \mathrm{Rel}_X \to \mathrm{Rel}_X$ is a relation $R \in \mathrm{Rel}_X$ with $R \subseteq g(R)$. Note that $\mathrm{Rel}_X$ is a complete lattice and that therefore any monotone map has a greatest post-fixpoint by the Knaster-Tarski Theorem [34]. We will define bisimulation, simulation, and duality as the greatest (post-)fixpoint of monotone functions, which we will therefore call *coinductive definitions*. This definition turns out to be intuitively what we would expect and the interaction of infinite behaviour with other type features is automatically correct. The coinductive definitions also give us immediately proof techniques for equivalence, duality and subtyping: to show that two states are, say, dual we only have to establish a relation that contains both states and show that the relation is a post-fixpoint. This technique can then be improved in various ways [30] and we will show that it is decidable for reasonable session coalgebras.

## 4.1 Bisimulation

Two states of a coalgebra are said to be *bisimilar* if they exhibit equivalent behaviour. We abstract away from the precise structure of a coalgebra and only consider its observable behaviour. Two states are bisimilar if their labels are equal and if the states at the end of matching transitions are again bisimilar. There is one exception to the equality of labels: basic types can be related via their pre-order, which does not have to coincide with equality.

Fix some coalgebra $(X, c)$ and let $c^* : \mathrm{Rel}_{F(X)} \to \mathrm{Rel}_X$ be the binary preimage of $c$ defined as

$$c^*(R) = \{(x, y) \mid (c(x), c(y)) \in R\}\,.$$

**Definition 5.** *We define the function* $f_\sim : \mathrm{Rel}_X \to \mathrm{Rel}_{F(X)}$ *as*

$$\begin{aligned} f_\sim(R) = \{\ ((a, f), (a, f')) \mid (\forall \alpha \in dom(f)) \quad f(\alpha)\ R\ f'(\alpha)\} \\ \cup \{\ ((\mathrm{bsc}, d, f_\emptyset), (\mathrm{bsc}, d', f_\emptyset)) \mid d \leq_D d' \wedge d' \leq_D d\ \} \end{aligned}$$

*where* $f_\emptyset \colon \emptyset \to X$ *is the empty function.*

It can be easily checked that, both, $c^*$ and $f_\sim$ are monotone maps and thus also their composition. Thus, the greatest fixpoint in the following definition exists.

**Definition 6.** *A relation $R$ is called a bisimulation if it is a post-fixpoint of* $c^* \circ f_\sim$. *We call the greatest fixpoint* bisimilarity *and denote it by* $\sim$.

### 4.2 Duality

Duality describes exactly opposite types in terms of their polarity. That is, the dual of input is output and the dual of output is input: $\overline{in} = out$ and $\overline{out} = in$. We can extend this to tuples $a$ in $A$, see Def. 3, with the exception of basic types because they do not describe channels:

$$\overline{(\text{com}, p)} = (\text{com}, \overline{p}) \qquad \overline{(\text{end})} = (\text{end})$$
$$\overline{(\text{branch}, p, L)} = (\text{branch}, \overline{p}, L) \qquad \overline{(\text{un})} = (\text{un})$$
$$\overline{(\text{bsc}, d)} \text{ is undefined}$$

The next step is to compare transitions. Continuations of $dom_C(f)$ need to be dual. The data types that are sent or received need to be equivalent, hence transitions of $dom_D(f)$ need to go to bisimilar states. We capture this idea with the monotone map $f_\perp : \text{Rel}_X \to \text{Rel}_{F(X)}$ defined as follows.

$$f_\perp(R) = \left\{ ((a, f), (\overline{a}, f')) \,\middle|\, \begin{array}{ll} (\forall \alpha \in dom_C(f)) & f(\alpha)\; R\; f'(\alpha) \text{ and} \\ (\forall \beta \in dom_D(f)) & f(\beta) \sim f'(\beta) \end{array} \right\}$$

**Definition 7.** *A relation $R$ is called a* duality relation *if it is a post-fixpoint of $c^* \circ f_\perp$. We call the greatest fixpoint* duality *and denote it by $\perp$.*

It is useful to have a function mapping any $x \in X$ to their dual $\overline{x}$, as long as duality is defined on $x$. However, even if duality is defined on $x$, the dual state might not be in $X$. Thus, we define the *dual closure* of $X$ as the set $X^\perp = X \cup \{\overline{x} \mid \overline{\sigma(x)} \text{ is defined}\}$, where $\overline{x}$ is understood to be an arbitrary state not in $X$ and distinct from $\overline{y}$ for any states $y \in X$ with $x \neq y$. For any of the original states, $c^\perp(x) = c(x)$, but for the new states we define $\sigma^\perp(\overline{x}) = \overline{\sigma(x)}$ and

$$\begin{array}{ll} \delta^\perp(\overline{x})(\alpha) = \overline{\delta(x)(\alpha)} & \text{for all } \alpha \in dom_C(f), \text{ and} \\ \delta^\perp(\overline{x})(\beta) = \delta(x)(\beta) & \text{for all } \beta \in dom_D(f) \end{array}$$

Thus, the dual closure is a coalgebra such that $x \perp \overline{x}$ for any $\overline{x}$. Notice that taking a dual twice always yields a bisimilar type, so we can define the duality function as an involution, $\overline{\overline{x}} = x$, rather than adding more variables. Clearly, the dual closure of a finite set is finite.

**Proposition 2.** *$x \perp \overline{x}$ for every state $x$ such that $\overline{x}$ is defined.*

### 4.3 Parallelizability

Unlike a linear endpoint, a channel endpoint with an unrestricted type may be shared between different parallel processes; each of them uses it independently, without informing the others. Furthermore, there is no way to coordinate which process receives which message. If the unrestricted endpoint sends a message, it could be read by a process that just started using the channel, or by a process that is almost done using the channel, or by a process that is anywhere in between.

In practice, this means an unrestricted channel can only perform one kind of communication action. However, session coalgebras allow us to define arbitrarily complex unrestricted types. For example, $\mu X.\ \mathtt{un}\,?\mathtt{int}.\ \mathtt{un}\,?\mathtt{bool}.\ X$ is an element of $\mathsf{Type}$, but we know that sending both `int` and `bool` over the same unrestricted channel causes problems.

**Definition 8.** *Given a coalgebra $(X, c)$, some subset $Y \subseteq X$ is* parallelizable*, written $par(Y)$, if for every $x$ and $y$ in $Y$ one of the following holds: $x \sim y$, $\sigma(x) = \mathrm{un}$, or $\sigma(y) = \mathrm{un}$.*

We know that un states do not represent communications; any other states, though, have to represent the same kind of action. We make this slightly stronger by requiring they are pairwise bisimilar.

Often we are interested in the parallelizability only of a specific state. Recall that $\langle x \rangle_c$ denotes the subcoalgebra generated by $x \in X$ in $c$.

**Definition 9.** *Let $\langle x \rangle_c^{\gg}$ be the smallest subset of $\langle x \rangle_c$ that contains $x$ and is closed under continuation transitions:*

$$\langle x \rangle_c^{\gg} = \bigcap \{ Y \subseteq X \mid x \in Y \ \textit{and}\ \delta(y)(\alpha) \in Y \ \textit{for all}\ y \in Y \ \textit{and}\ \alpha \in dom_C(\delta(y)) \ \}$$

*A state $x$ is parallelizable, written $par(x)$, if $\langle x \rangle_c^{\gg}$ is parallelizable.*

### 4.4 Simulation and Subtyping

Intuitively, a coalgebra simulates another if the behaviour of the latter "is contained in" the former. Subtyping, originally defined on session types by Gay and Hole [17], is a notion of substitutability of types [16]. We will define our notion of simulation such that it coincides with subtyping, just like bisimulation provides a notion of type equivalence [17].

Consider a process that expects a channel of type $T = ?\mathtt{real}$. The process reads a value, and expects it to be a real number and treat it as such. We defined `int` as a subtype of `real`, so the process can operate correctly if it receives an integer instead; that is, $?\mathtt{int}$ is a subtype of $T$. Now consider a process that expects a channel of type $!\mathtt{int}$, on which it can send any integer. In this case we cannot restrict the channel to a subtype: as all integers are valid where real numbers are expected, we can generalise the channel type to $!\mathtt{real}$.

Now, in the input case the session types are related (in the subtyping relation) in the same order as the data types; this is called *covariance*. For output, the order is reversed; this is called *contravariance*. The same idea holds for labelled choices: the subtype of an external choice can have a subset of choices, while the subtype of an internal choice can add more options. For all types, it holds that states reached through transitions are covariant, i.e., if $T$ is a subtype of $U$, continuations of $T$ must be subtypes of continuations (of the same label) of $U$. The monotone map $h_{\sqsubseteq}$ in Fig. 7 captures these ideas formally.

**Definition 10.** *A relation $R$ is called a* simulation *if it is a post-fixpoint of $c^* \circ h_{\sqsubseteq}$. We call the greatest fixpoint* similarity *and denote it by $\sqsubseteq$.*

$$
\begin{aligned}
h_{\sqsubseteq}(R) = \{\, &((\mathrm{com}, \mathit{in}, f), (\mathrm{com}, \mathit{in}, g)) &&\mid f(*) \mathrel{R} g(*) \text{ and } f(\not{d}) \mathrel{R} g(\not{d}) \,\} \\
\cup \{\, &((\mathrm{com}, \mathit{out}, f), (\mathrm{com}, \mathit{out}, g)) &&\mid f(*) \mathrel{R} g(*) \text{ and } g(\not{d}) \mathrel{R} f(\not{d}) \,\} \\
\cup \{\, &((\mathrm{branch}, \mathit{in}, L_1, f), \\
&\qquad (\mathrm{branch}, \mathit{in}, L_2, g)) &&\mid L_1 \subseteq L_2 \text{ and } \forall l \in L_1.\ f(l) \mathrel{R} g(l) \,\} \\
\cup \{\, &((\mathrm{branch}, \mathit{out}, L_1, f), \\
&\qquad (\mathrm{branch}, \mathit{out}, L_2, g)) &&\mid L_2 \subseteq L_1 \text{ and } \forall l \in L_2.\ f(l) \mathrel{R} g(l) \,\} \\
\cup \{\, &((\mathrm{bsc}, d, f_\emptyset), (\mathrm{bsc}, d', f_\emptyset)) &&\mid d \leq_D d' \,\} \\
\cup \{\, &((\mathrm{end}, f_\emptyset), (\mathrm{end}, f_\emptyset)) \,\} \\
\cup \{\, &((\mathrm{un}, f), (\mathrm{un}, g)) &&\mid f(*) \mathrel{R} g(*), \text{ and } \mathrm{par}(f(*)) \text{ iff } \mathrm{par}(g(*)) \,\}
\end{aligned}
$$

**Figure 7.** Monotone map $h_{\sqsubseteq} \colon \mathrm{Rel}_X \to \mathrm{Rel}_{F(X)}$ that defines simulations

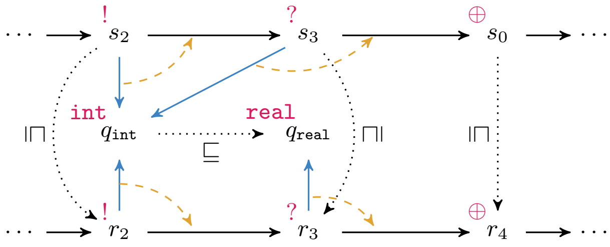

**Figure 8.** Simulation for two mathematical server clients (indicated by dotted arrows)

Let us illustrate similarity by means of an example.

*Example 1.* Recall the two client protocols for our mathematical server in Figs. 2 and 3. We can now prove our claim that the latter can also connect to the server because it is a supertype of the client protocol in Fig. 2. To do that, we have to establish a simulation relation between the states of both client protocols. In Fig. 8, we display a part of both session coalgebras side-by-side and indicate with dotted arrows the pairs that have to be related by a simulation relation to show that these states are similar, that is, related by $\sqsubseteq$. It should be noted that we simulate states from the second coalgebra by that of the first, that is, we show $s_k \sqsubseteq r_k$ for the shown states. There is one exception to this, namely $q_{\mathtt{int}} \sqsubseteq q_{\mathtt{real}}$.

The following proposition records some properties of and tight connections between the relations that we introduced.

**Proposition 3.** *Bisimilarity $\sim$ is an equivalence relation, duality $\perp$ is symmetric, and similarity $\sqsubseteq$ is a preorder. Moreover, for all states $x$, $y$, and $z$ of a session coalgebra, we have that*

1. *$x \sim y$ iff $x \sqsubseteq y$ and $y \sqsubseteq x$;*
2. *$x \perp y$ and $x \perp z$ implies $y \sim z$; and*
3. *$x \perp y$ and $y \sim z$ implies $x \perp z$ .*

### 4.5 Decidability

In a practical type checker, we need an algorithm to decide the relations defined above. In this subsection we show an algorithm that computes the answer in finite time for a certain class of types.

**Definition 11.** *A coalgebra $c$ is* finitely generated *if $\langle x \rangle_c$ is finite for all $x$.*

This restriction is not problematic for types, as the following lemma shows.

**Lemma 1.** *The coalgebra of types $(\mathsf{Type}, c_{\mathsf{Type}})$ is finitely generated.*

To determine whether two states $x$ and $y$ are bisimilar, we need to determine if there exists a bisimulation $R$ with $x\,R\,y$. We start with the simplest relation $R = \{(x, y)\}$, and ask if this is a bisimulation.

First, we check that for all $(u, w) \in R$, $\sigma(u) = \sigma(w)$, or in the case of bsc states that $\mathrm{da}(u) \leq_D \mathrm{da}(w)$ and $\mathrm{da}(w) \leq_D \mathrm{da}(u)$. If $\sigma(u) \neq \sigma(w)$ for any pair in $R$ we know that no superset of $R$ is a bisimulation, and the algorithm rejects.

Second, we check the matching transitions. For every $(u, w) \in R$ and $\alpha \in dom(\delta(u))$ we check whether $(\delta(u)(\alpha), \delta(w)(\alpha)) \in R$. If we encounter a missing pair, we add it to $R$ and ask whether this new relation is a bisimulation, i.e., return to the first step. If all destinations for matching transitions are present in $R$, then $R$ is, by construction, a bisimulation containing $(x, y)$. Hence, $x \sim y$.

This algorithm tries to construct the smallest possible bisimulation containing $(x, y)$, by only adding strictly necessary pairs. If the algorithm rejects, there is no such bisimulation; hence, $x \not\sim y$. Additionally, the algorithm only examines pairs in $\langle x \rangle_c \times \langle y \rangle_c$. If there are finitely many of such pairs, the algorithm will terminate in finite time

The above described algorithm can be suitably adapted to similarity and duality, which gives us the following result.

**Theorem 1.** *Bisimilarity, similarity, and duality of any states $x$ and $y$ are decidable if $\langle x \rangle_c$ and $\langle y \rangle_c$ are finite. Parallelizability of any state $x$ is decidable if $\langle x \rangle_c^{\gg}$ (Definition 9) is finite.*

**Corollary 1.** *Bisimilarity, similarity, and duality are decidable for $c_{\mathsf{Type}}$.*

## 5 Typing Rules

Session types are meant to discipline the behaviour of the channels of an interacting process, so as to ensure that prescribed protocols are executed as intended. Up to here, we have focused on session types (i.e., their representation as session coalgebras and coinductively-defined relations on them) without committing to a specific syntax for processes. This choice is on purpose: our goal is to provide a truly syntax-independent justification for session types. In this section, we introduce a syntactic notion of processes and rely on session coalgebras to define the typing rules for a session type system.

| | | |
|---|---|---|
| $P, Q ::= \overline{x}\langle y\rangle.P$ | | output $y$ on channel $x$ |
| $\mid$ | $x(y).P$ | bind input from channel $x$ to variable $y$ |
| $\mid$ | $x \triangleright \{l_i : P_i\}_{i\in I}$ | offer choices $l_1, l_2, \ldots$ |
| $\mid$ | $x \triangleleft l.P$ | make choice $l$ |
| $\mid$ | $P \mid Q$ | composition |
| $\mid$ | $!P$ | replication |
| $\mid$ | $\mathbf{0}$ | finished process |
| $\mid$ | $(\nu xy)P$ | channel creation |

**Figure 9.** Process syntax

### 5.1 A Session $\pi$-calculus

The $\pi$-calculus is a formal model of interactive computation in which processes exchange messages along channels (or names) [26,31]. As such, it is an abstract framework in which key features such as name mobility, (message-passing) concurrency, non-determinism, synchronous communication, and infinite behaviour have rigorous syntactic representations and precise operational meaning. We consider a *session* $\pi$-calculus based on [37,17], i.e., a variant of the $\pi$-calculus whose operators are tailored to the protocols expressed by session types.

We assume base sets of *variables* $(x, y, z, \ldots)$ and *values* $(v, v', \ldots)$, which can be variables or the Boolean constants (true and false). There is also a set of *labels* $\mathbb{L}$, ranged over by $l, l', \ldots$. The syntax of processes $(P, Q, \ldots)$ is given by the grammar in Fig. 9. We discuss the salient aspects of the syntax. A process $\overline{x}\langle y\rangle.P$ denotes the output of channel $y$ along channel $x$, which precedes the execution of $P$. Dually, a process $x(y).P$ denotes the input of a value $v$ along channel $x$, which precedes the execution of process $P[v/y]$, i.e., the process $P$ in which all free occurrences of $y$ have been substituted by $v$. Processes $x \triangleright \{l_i : P_i\}_{i\in I}$ and $x \triangleleft l.P$ implement a labelled choice mechanism. Given a finite index set $I$, process $x \triangleright \{l_i : P_i\}_{i\in I}$, known as branching, denotes an external choice: the reception of a label $l_j$ (with $j \in I$) along channel $x$ precedes the execution of the continuation $P_j$. Process $x \triangleleft l.P$, known as selection, denotes an internal choice; it is meant to interact with a complementary branching. Given processes $P$ and $Q$, process $P \mid Q$ denotes their parallel composition, which enables their simultaneous execution. The process $!P$, the replication of $P$, denotes the composition of infinite copies of $P$ running in parallel, i.e., $P \mid P \mid \cdots$. Process $\mathbf{0}$ denotes inaction. Finally, process $(\nu xy)P$ is arguably the main difference with respect to usual presentations of the $\pi$-calculus, and denotes a restriction operator that declares $x$ and $y$ as *covariables*, i.e., as complementary endpoints of the same channel, with scope $P$.

The operational semantics for processes is defined as a *reduction relation* denoted $\longrightarrow$, by relying on a notion of *structural congruence* on processes, denoted $\equiv$. Figure 10 defines these two notions. Intuitively, two processes are structurally congruent if they are identical in behaviour, but not necessarily in structure. It is the smallest congruence relation satisfying the axioms in Fig. 10 (bottom). We say a process $P$ reduces to $Q$, written $P \longrightarrow Q$, when there is a single execution step yielding $Q$ from $P$. We comment on the rules in Fig. 10 (top). R-COM formalizes

**Reduction**

$$(\nu xy)(\overline{x}\langle v\rangle.P \mid y(z).Q \mid R) \longrightarrow (\nu xy)(P \mid Q[v/z]|R) \qquad \text{[R-COM]}$$

$$(\nu xy)(x \triangleleft l_j.P \mid y \triangleright \{l_i : Q_i\}_{i\in I} \mid R) \longrightarrow (\nu xy)(P \mid Q_j \mid R) \quad (j \in I) \qquad \text{[R-SYNC]}$$

$$\frac{P \longrightarrow Q}{(\nu xy)P \longrightarrow (\nu xy)Q} \qquad \frac{P \longrightarrow Q}{P \mid R \longrightarrow Q \mid R} \qquad \text{[R-RES][R-PAR]}$$

$$\frac{P \equiv P' \quad P \longrightarrow Q \quad Q \equiv Q'}{P' \longrightarrow Q'} \qquad \text{[R-CONG]}$$

**Structural congruence**

*Parallel composition:*

$$P \mid Q \equiv Q \mid P \qquad (P \mid Q) \mid R \equiv P \mid (Q \mid R) \qquad P \mid \mathbf{0} \equiv P \qquad !P \equiv P \mid !P$$

*Scope restriction:*

$$(\nu xy)(\nu vw)P \equiv (\nu vw)(\nu xy)P \qquad (\nu xy)\mathbf{0} \equiv \mathbf{0} \qquad (\nu xy)P \equiv (\nu yx)P$$

$$(\nu xy)(P \mid Q) \equiv ((\nu xy)P) \mid Q \qquad \text{if } x \text{ and } y \text{ not free in } Q$$

**Figure 10.** Reduction semantics the exchange a value over a channel formed by two covariables. Similarly, R-SYNC formalises the synchronisation between a branching and a selection that realises the labelled choice. Rules R-RES and R-PAR are contextual rules, which allow reduction to proceed under restriction and parallel composition. Finally, Rule R-CONG says that reduction is closed under structurally congruence: we can use $\equiv$ to promote interactions that match the structure of the rules above.

## 5.2 Typing Rules

Based on the above, variables $P, Q$ will refer to processes, $x, y, z$ will range over channels and $T, U, V$ are states of some fixed, but arbitrary, session coalgebra $(X, c)$. Variables are associated with these states in a *context* $\Gamma$, as described by $\Gamma ::= \emptyset \mid \Gamma, x : T$ . A context is an unordered, finite set of pairs, that may have at most one pair $(x, T)$ for each variable $x$. A context is thus isomorphic to a (partial) function from a finite set of variables to their types. We use $\Gamma$ to denote this isomorphic function as well: $\Gamma(x) = T$ if $(x, T) \in \Gamma$. The domain of a context is defined accordingly.

We know 'un' types are unrestricted, but they are not the only ones.

**Definition 12.** *A type is* unrestricted*, written un*$(T)$*, if its operation is* un*,* end *or* bsc*. A context is unrestricted, written un*$(\Gamma)$*, if all types in* $\Gamma$ *are unrestricted, i.e., if* $(x, T) \in \Gamma$ *implies un*$(T)$*. A type is* linear*, written lin*$(T)$*, if it is not unrestricted. A context is linear, if all its types are linear.*

A context $\Gamma$ may be split into two parts $\Gamma_1$ and $\Gamma_2$, such that the linear types are strictly divided between $\Gamma_1$ and $\Gamma_2$, but unrestricted types can be copied. Context split is a ternary relation, defined by the axioms in Fig. 11. We may write $\Gamma_1 \circ \Gamma_2$ to refer to a context $\Gamma$ for which $\Gamma = \Gamma_1 \circ \Gamma_2$ is in the context split relation. Such a context is not necessarily defined for any given contexts;

$$\emptyset = \emptyset \circ \emptyset \qquad \frac{\Gamma = \Gamma_1 \circ \Gamma_2 \qquad \mathrm{un}(T)}{\Gamma, x : T = (\Gamma_1, x : T) \circ (\Gamma_2, x : T)}$$

$$\frac{\Gamma = \Gamma_1 \circ \Gamma_2}{\Gamma, x : T = (\Gamma_1, x : T) \circ \Gamma_2} \qquad \frac{\Gamma = \Gamma_1 \circ \Gamma_2}{\Gamma, x : T = \Gamma_1 \circ (\Gamma_2, x : T)}$$

**Figure 11.** Context Split

$$\frac{\mathrm{un}(\Gamma)}{\Gamma \vdash \mathbf{0}} \qquad \frac{\Gamma, x : T, y : U \vdash P \qquad T \perp U}{\Gamma \vdash (\nu xy)P} \qquad \text{[T-Inact][T-Res]}$$

$$\frac{\Gamma_1 \vdash P \qquad \Gamma_2 \vdash Q}{\Gamma_1 \circ \Gamma_2 \vdash P \mid Q} \qquad \frac{\Gamma \vdash P \qquad \mathrm{un}(\Gamma)}{\Gamma \vdash !P} \qquad \text{[T-Par][T-Rep]}$$

$$\frac{c(T) = (?, f) \qquad \Gamma,\, y : U,\, x : f(*) \vdash P \qquad f(\not{d}) \sqsubseteq U}{\Gamma, x : T \vdash x(y).P} \qquad \text{[T-In]}$$

$$\frac{c(T) = (!, f) \qquad \Gamma,\, x : f(*) \vdash P \qquad U \sqsubseteq f(\not{d})}{\Gamma, x : T, y : U \vdash \overline{x}\langle y \rangle.P} \qquad \text{[T-Out]}$$

$$\frac{c(T) = (\&, L_1, f) \qquad L_1 \subseteq L_2 \qquad \Gamma, x : f(l) \vdash P_l \qquad \forall l \in L_1}{\Gamma, x : T \vdash x \triangleright \{l : P_l\}_{l \in L_2}} \qquad \text{[T-Branch]}$$

$$\frac{c(T) = (\oplus, L, f) \qquad \Gamma, x : f(l) \vdash P_l \qquad l \in L}{\Gamma, x : T \vdash x \triangleleft l.P_l} \qquad \text{[T-Sel]}$$

$$\frac{c(T) = (\mathrm{un}, f) \qquad \mathrm{par}(T) \qquad \Gamma, x : f(*) \vdash P}{\Gamma, x : T \vdash P} \qquad \text{[T-Unpack]}$$

**Figure 12.** Declarative Typing Rules we implicitly assume its existence when writing $\Gamma_1 \circ \Gamma_2$. Notice that the use of $\Gamma, x : T$ in the third rule of Fig. 11 carries the assumption that $x$ not in $\Gamma$. Otherwise, $\Gamma, x : T$ would have two pairs with $x$, which is not allowed.

The type system is defined by the rules in Fig. 12. A process $P$ is *well-formed*, under a context $\Gamma$, if there is some inference tree whose root is $\Gamma \vdash P$ and whose nodes are all valid instantiations of these type rules. As T-Inact is the only rule that does not depend on the correctness of another process, it forms the leaves of such trees. For well-formed processes, the type system guarantees that:

- If the process terminates, then all linear sessions were completed.
- If a process reads a value from a channel, the value has the type specified by the channel's session type. If a process receives a label, it is one of the labels specified by the channel's session type.

We discuss the typing rules, which can be conveniently read keeping in mind the notations introduced in Def. 3 and Prop. 1. T-Inact ensures that all linear channels in the context are interacted with until the type becomes unrestricted. If our context contains a variable $x$ of type $?\texttt{int}$, then the process is required to read an `int` from it. Thus, $x : ?\texttt{int}. \nvdash \mathbf{0}$. In contrast, process $x(z).\mathbf{0}$ is well-formed for the same context, using T-Inact and T-In:

$$\frac{\overline{x : \text{end}, z : \texttt{int} \vdash \mathbf{0}}}{x : ?\texttt{int} \vdash x(z).\mathbf{0}}$$

T-Res creates a channel by binding together two covariables $x$ and $y$, of dual type. T-Par causes unrestricted channels to be copied and linear channels to get split between composite processes, ensuring the latter occur in only a single process. Recall that replication $!P$ is an infinite composition of a single process $P$, hence, a replicated process can only use unrestricted channels. Together, T-Par and T-Res allow us to introduce new covariables, with new types, and distribute them. But, only unrestricted types may be copied. Notice that a process does not specify which types to give the newly bound variables.

$$\begin{array}{rcl} v : \texttt{int} & \vdash & (\nu xy)\, x(z).\mathbf{0} \mid \overline{y}\langle v\rangle.\mathbf{0} \\ x : un?\texttt{int} & \vdash & x(z).\mathbf{0} \mid x(z).\mathbf{0} \\ x :?\texttt{int} & \nvdash & x(z).\mathbf{0} \mid x(z).\mathbf{0} \end{array}$$

Each action on a channel has its own rule: T-In handles input, binding the channel $x$ to the continuation type and $y$ to some supertype of the received type. T-Out handles output, which requires the sent variable to have a subtype of whatever type the channel expects to send. T-Branch handles external choice, where the process needs to offer at least all choices the type describes, coupled with processes that are correctly typed under the respective continuation types. T-Sel only checks whether the single label that was chosen by the process was a valid option, and if the rest of the process is correct under the continuation type.

These rules are only specified for linear states; T-Unpack allows a un state to be used as if it was the underlying type, as long as it is parallelizable (Def. 8).

We can actually create structures with un that do not have a syntactical equivalent. For example, let $T_{\text{end}}$ be a state with $\sigma(T_{\text{end}}) = \text{un}$ and $\delta(T_{\text{end}})(*) = T_{\text{end}}$. Just like regular end, $T_{\text{end}}$ allows no interactions on the channel, but it does not cause a "un" type to be unparallelizable.

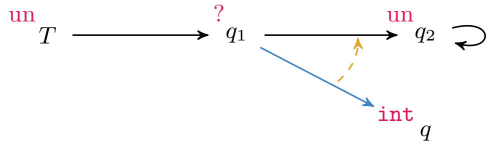

**Figure 13.** Session coalgebra using an alternative completed protocol

The diagram in Fig. 13 describes a parallelizable unrestricted state $T$ such that each copy of a channel in state $T$ can only do a single receive. However, because it is unrestricted, we can still copy the channel across threads and read a value per copy. We can even read infinitely many values through replication.

$$\begin{array}{lll} x : T & \nvdash & x(y_1).x(y_2).x(y_3).\mathbf{0} \\ x : T & \vdash & x(y_1).\mathbf{0} \mid x(y_2).\mathbf{0} \mid x(y_3).\mathbf{0} \\ x : T & \vdash & !(x(y).\mathbf{0}) \end{array}$$

Such a type might be interesting in combination with session delegation. A linear session could be established by receiving a channel from an unrestricted channel. By using a structure like $T$, each thread is guaranteed to establish at most one private session, but there can be many of such sessions in parallel threads.

In Sec. 4, we defined simulation through the intuition of subtyping as substitutability in one direction. We see that substitution is indeed allowed for simulated types.

**Theorem 2.** *The following, more common, rule is admissible from the rules in Fig. 12.*

$$\frac{\Gamma, x : T \vdash P \qquad U \sqsubseteq T}{\Gamma, x : U \vdash P}$$

That is, we could add the rule as an axiom, without changing the set of typable processes. As a corollary, bisimulation of states implies the states are equivalent with respect to the type system.

**Corollary 2.** *For all bisimilar types $T \sim U$, contexts $\Gamma$ and processes $P$, it holds that $\Gamma, x : T \vdash P$ if and only if $\Gamma, x : U \vdash P$.*

## 6 Algorithmic Type Checking

The type rules describe what well-formed processes look like, but do not directly allow us to decide whether an arbitrary process is well-formed or not. This is because, beforehand, we do not know:

1. Which type to introduce in reading (T-In) or scope restriction (T-Res), or
2. How to split the context in composite processes (T-Par).

Rather than trying to infer the introduced types, we augment the language of processes with type annotations:

$$P ::= \ldots \mid (\nu xy : T)\, P \mid x(y : T).P$$

We only need to annotate one type for scope restrictions, as we can create the other with the duality function. Other productions are kept unchanged.

When checking a process $P \mid Q$, we pass along the entire context to $P$, keeping track of all linear variables used, and remove those from the context given to $Q$. To do this we add an *output* to the algorithm: in an execution $\Gamma_1 \vdash P \,;\, \Gamma_2$, output $\Gamma_2$ is the subset of $\Gamma_1$ containing only those variables of the input which

$$\Gamma \div \emptyset = \Gamma \qquad \frac{\Gamma_1 \div F = \Gamma_2, x : T \quad \mathrm{un}(T)}{\Gamma_1 \div (F, x) = \Gamma_2} \qquad \frac{\Gamma_1 \div F = \Gamma_2 \quad x \notin dom(\Gamma_2)}{\Gamma_1 \div (F, x) = \Gamma_2}$$

**Figure 14.** Context Difference

$$\Gamma \vdash \mathbf{0}; \Gamma \qquad \frac{\Gamma_1 \vdash P; \Gamma_2 \quad \Gamma_1 = \Gamma_2}{\Gamma_1 \vdash\, !P; \Gamma_2} \qquad \text{[A-INACT][A-REP]}$$

$$\frac{\Gamma_1 \vdash P \;;\; \Gamma_2 \quad \Gamma_2 \vdash Q \;;\; \Gamma_3}{\Gamma_1 \vdash P \mid Q \;;\; \Gamma_3} \qquad \frac{\Gamma_1, x : T, y : \overline{T} \vdash P; \Gamma_2}{\Gamma_1 \vdash (\nu xy : T)P \;;\; \Gamma_2 \div \{x, y\}} \qquad \text{[A-PAR][A-RES]}$$

$$\frac{c(T) = (?, f) \quad f(d) \sqsubseteq U \quad \Gamma_1, y : U, x : f(*) \vdash P \;;\; \Gamma_2}{\Gamma_1, x : T \vdash x(y : U).P \;;\; \Gamma_2 \div \{x, y\}} \qquad \text{[A-IN]}$$

$$\frac{c(T) = (!, f) \quad U \sqsubseteq f(d) \quad \Gamma_1, x : f(*) \vdash P \;;\; \Gamma_2}{\Gamma_1, x : T, y : U \vdash \overline{x}\langle y \rangle.P \;;\; \Gamma_2 \div \{x\}} \qquad \text{[A-OUT]}$$

$$\text{[A-BRANCH]}$$

$$\frac{c(T) = (\&, L_1, f) \quad L_1 \subseteq L_2 \quad \Gamma_1, x : f(l) \vdash P_l \;;\; \Gamma_l \quad \Gamma_2 = \Gamma_l \div \{x\} \quad \forall l \in L_2}{\Gamma_1, x : T \vdash x \rhd \{l : P_l\}_{l \in L_2} \;;\; \Gamma_2}$$

$$\frac{c(T) = (\oplus, L, f) \quad \Gamma_1, x : f(l) \vdash P_l \;;\; \Gamma_2 \quad l \in L}{\Gamma_1, x : T \vdash x \lhd l.P_l \;;\; \Gamma_2 \div \{x\}} \qquad \text{[A-SEL]}$$

$$\frac{c(T) = (\mathrm{un}, f) \quad \mathrm{par}(T) \quad \Gamma_1, x : f(*) \vdash P; \Gamma_2}{\Gamma_1, x : T \vdash P \;;\; (\Gamma_2 \div \{x\}), x : T} \qquad \text{[A-UNPACK]}$$

**Figure 15.** Algorithmic Type Checking Rules had unrestricted types or were not used in $P$. We say subset because we want these variables, if present, to have the same type in $\Gamma_2$ as in $\Gamma_1$.

Figure 15 lists the algorithmic versions of the type rules. A-PAR, for example, checks parallel processes as described. By construction, $\Gamma_2$ is one part of the context split required to instantiate T-PAR. The linear variables of the other part is exactly those which are present in $\Gamma_1$ but not in $\Gamma_2$. This change in A-PAR requires adjusting the other rules. Firstly, we need the algorithm to accept even when we do not fully complete all sessions of $\Gamma_1$ in $P$. We do this by unconditionally accepting the terminated process. Note that acceptance of the algorithm now only implies well-formedness if the returned context is unrestricted.

Secondly, the algorithm needs to remove linear variables from the output as we use them. We do not, however, want to remove any variable that has a linear type, as that would allow us to accept processes which do not complete all linear sessions. Thus, we introduce the context difference operator $\div$ in Fig. 14. $\Gamma \div \{x\}$ is the context of all variable/type pairs in $\Gamma$ minus a potential pair including $x$, but is only defined if $(x, T) \in \Gamma$ implies that $T$ is unrestricted.

We elaborate on A-Branch; the algorithm is called once for every branch, yielding a context $\Gamma_l$ each time. Excluding $x$, each branch must use the exact same set of linear variables. Thus, we require that all these contexts are equal up to a potential $(x, U_l)$ pair. Specifically, there is some $\Gamma_2$ such that $\Gamma_2 = \Gamma_l \div \{x\}$ for any $l \in L_2$, this $\Gamma_2$ is the output context.

To motivate this, consider a type $T = \&\{a : T_{un},\, b : \text{end}\}$, where $T_{un}$ is some unrestricted type distinct from end, and some process $P = x \triangleright \{a : \mathbf{0},\, b : \mathbf{0}\}$. Let $\Gamma$ be some unrestricted context, $\mathbf{0}$ is well-formed for both $\Gamma, x : T_{un}$ and $\Gamma, x : \text{end}$; the algorithm agrees.

$$\Gamma, x : T_{un} \vdash \mathbf{0} \;;\; (\Gamma, x : T_{un})$$
$$\Gamma, x : \text{end} \vdash \mathbf{0} \;;\; (\Gamma, x : \text{end})$$

The resulting contexts are not equal. $P$ is well-formed for $\Gamma$, so we have to allow $x$ to have different types in the output of different branches in a complete algorithm. A-In, A-Out, and A-Sel do not have multiple branches to check, but the ideas are similar. When introducing a new variable, either through a read or scope restriction, the new variable is also removed from the output. A-Unpack only unpacks unrestricted types. We want those to have the same type in the input as in the output, so we remove the variable and add a pair with the original type.

Take, for example, the process

$$x : ?\texttt{int},\; y : ?\texttt{int} \quad \vdash \quad x(z_1).\mathbf{0} \mid y(z_2).\mathbf{0}$$

The variables are split correctly, and both split contexts are unrestricted when the process is completed, thus it is well-formed.

If, on the other hand, the left process did not complete the linear session, then the context difference would not have been defined. Take one such process:

$$x : ?\texttt{int}.?\texttt{int},\; y : ?\texttt{int} \quad \nvdash \quad x(z_1).\mathbf{0} \mid y(z_2).\mathbf{0}$$

We succeed in checking the terminated process of the left part.

$$x : ?\texttt{int},\; y : ?\texttt{int} \quad \vdash \quad \mathbf{0}\;; \quad (x : ?\texttt{int}, y : ?\texttt{int})$$

But $x$ has a linear type in the output. $(x : ?\texttt{int}, y : ?\texttt{int}) \div \{x\}$ is undefined, so the algorithm rejects this input entirely. The process was indeed not well-formed, and no further parallel processes could fix it; the rejection is expected.

For each process and context there is at most one applicable algorithmic rule: which one is directed by the process syntax and unrestrictedness of a channel being interacted with.

Under the same assumptions as before (i.e., the session coalgebra describing the types is finitely generated), this induced type checking algorithm is decidable, sound, and complete with respect to the type rules defined in Sec. 5.

**Theorem 3 (Decidability).** *The type checking algorithm terminates in finite time for every input, assuming a finitely generated session coalgebra.*

Having defined algorithmic typechecking, we can go back to the language that we used to define our typing rules by erasing type annotations in input and restriction operators. Let $erase(\cdot)$ denote a function on processes defined as

$$erase((\nu xy : T).Q) = (\nu xy).erase(Q)$$
$$erase(x(y : T).Q) = x(y).erase(Q)$$

and as an homomorphism on the remaining process constructs. We have:

**Theorem 4 (Correctness).** *For any context $\Gamma$ and annotated process $P$, $\Gamma_1 \vdash erase(P)$ iff $\Gamma_1 \vdash P; \Gamma_2$ and $un(\Gamma_2)$.*

## 7 Concluding Remarks

We have developed a new, language-independent foundation for session types by relying on coalgebras. We introduced session coalgebras, which elegantly capture all communication structures of session types, both linear and unrestricted, without committing to a specific syntactic formulation for processes and types. Session coalgebras allow us to rediscover language-independent coinductive definitions for duality, subtyping, and type equivalence. A key idea is to assimilate channel types to the states of a session coalgebra; we demonstrated this insight by deriving a session type system for the $\pi$-calculus, which revisits and extends that by Vasconcelos [37], unlocking decidability results and algorithmic type checking.

Interesting strands for future work include extending our coalgebraic toolbox so as to give a language-independent justification to advanced session type systems, such as context-free session types [35] and multiparty session types [21]. Another line concerns extending our coalgebraic view to include *language-dependent* issues and properties that require a global analysis on session behaviours. Salient examples are *liveness* properties such as (dead)lock-freedom and progress: advanced type systems [23,29,28,8] typically couple (session) types with advanced mechanisms (such as priority-based annotations and strict partial orders), which provide a global insight to rule out the circular dependencies between sessions that are at the heart of stuck processes. Lastly, the whole area of coalgebra now becomes available to explore session types. One possible direction is to make use of final coalgebras and modal logic, which would allow us to analyse the behaviour of session coalgebras. This would be particularly powerful in combination with composition operations for session coalgebras to break down protocols and type checking. Another direction is to use session coalgebras to verify other coalgebras that take on the role of the syntactic $\pi$-calculus [12,27] and thereby allowing also for the exploration of other semantics like manifest sharing [1,2] without resorting to a specific syntax.

*Acknowledgements* We are grateful to the anonymous reviewers for their useful remarks and suggestions. Pérez has been partially supported by the Dutch Research Council (NWO) under project No. 016.Vidi.189.046 (Unifying Correctness for Communicating Software).
